# Revisiting spatial distribution and biochemical composition of calcium-containing crystals in human osteoarthritic articular cartilage

**DOI:** 10.1186/ar4283

**Published:** 2013-09-03

**Authors:** Christelle Nguyen, Dominique Bazin, Michel Daudon, Aurore Chatron-Colliet, Didier Hannouche, Arnaud Bianchi, Dominique Côme, Alexander So, Nathalie Busso, Frédéric Lioté, Hang-Korng Ea

**Affiliations:** 1 Université Paris Diderot, Sorbonne Paris Cité, UFR de Médecine, F-75205 Paris, France; 2 INSERM, UMR-S606, hôpital Lariboisière, F-75475 Paris, France; 3 CNRS-LCMCP-UPMC, Collège de France, 11 place M. Berthelot, 75231 Paris Cedex 05, France; 4 Service des Explorations Fonctionnelles, Hôpital Tenon, Assistance Publique-Hôpitaux de Paris, 75020 Paris, France; 5 Service de chirurgie orthopédique, Hôpital Lariboisière, Assistance Publique-Hôpitaux de Paris, Université Paris Denis Diderot, Faculty of Medicine, 75010 Paris, France; 6 Ingénierie Moléculaire et Physiopathologie Articulaire (IMoPA), UMR 7365 CNRS-Université de Lorraine, Faculté de medicine, Vandoeuvre Lès Nancy, 54505, France; 7 DAL, Service de Rhumatologie, Centre Hospitalier Universitaire Vaudois et Université de Lausanne, CH-1011, Lausanne, Switzerland; 8 Assistance Publique-Hôpitaux de Paris, Hôpital Lariboisière, Service de Rhumatologie, Centre Viggo Petersen, Pôle Appareil Locomoteur, 2 Rue Ambroise Paré, F-75010 Paris, France

## Abstract

**Introduction:**

Calcium-containing (CaC) crystals, including basic calcium phosphate (BCP) and calcium pyrophosphate dihydrate (CPP), are associated with destructive forms of osteoarthritis (OA). We assessed their distribution and biochemical and morphologic features in human knee OA cartilage.

**Methods:**

We prospectively included 20 patients who underwent total knee replacement (TKR) for primary OA. CaC crystal characterization and identification involved Fourier-transform infra-red spectrometry and scanning electron microscopy of 8 to 10 cartilage zones of each knee, including medial and lateral femoral condyles and tibial plateaux and the intercondyle zone. Differential expression of genes involved in the mineralization process between cartilage with and without calcification was assessed in samples from 8 different patients by RT-PCR. Immunohistochemistry and histology studies were performed in 6 different patients.

**Results:**

Mean (SEM) age and body mass index of patients at the time of TKR was 74.6 (1.7) years and 28.1 (1.6) kg/m², respectively. Preoperative X-rays showed joint calcifications (chondrocalcinosis) in 4 cases only. The medial femoro-tibial compartment was the most severely affected in all cases, and mean (SEM) Kellgren-Lawrence score was 3.8 (0.1). All 20 OA cartilages showed CaC crystals. The mineral content represented 7.7% (8.1%) of the cartilage weight. All patients showed BCP crystals, which were associated with CPP crystals for 8 joints. CaC crystals were present in all knee joint compartments and in a mean of 4.6 (1.7) of the 8 studied areas. Crystal content was similar between superficial and deep layers and between medial and femoral compartments. BCP samples showed spherical structures, typical of biological apatite, and CPP samples showed rod-shaped or cubic structures. The expression of several genes involved in mineralization, including human homolog of progressive ankylosis, plasma-cell-membrane glycoprotein 1 and tissue-nonspecific alkaline phosphatase, was upregulated in OA chondrocytes isolated from CaC crystal-containing cartilages.

**Conclusions:**

CaC crystal deposition is a widespread phenomenon in human OA articular cartilage involving the entire knee cartilage including macroscopically normal and less weight-bearing zones. Cartilage calcification is associated with altered expression of genes involved in the mineralisation process.

## Introduction

Osteoarthritis (OA) is a whole-joint disease characterized by cartilage destruction with cartilage mineralization, subchondral bone modifications and mild synovial inflammation. The two main types of calcium-containing (CaC) crystals encountered in hyaline and fibrous cartilage mineralization are calcium pyrophosphate (CPP) and basic calcium phosphate (BCP) crystals. Cartilage and meniscus calcification evidenced on conventional radiographs is named chondrocalcinosis [1]. BCP crystals are a heterogeneous group of CaC crystals that encompass hydroxyapatite, carbonated apatite (CA), octacalcium phosphate, amorphous carbonated calcium phosphate (ACCP), tricalcium phosphate and whitlockite (WH) (magnesium-substituted) crystals. Both monoclinic and triclinic CPP crystals and the different members of BCP crystals have been found in synovial fluid [2]. The pathogenic role of CPP and BCP crystal deposition in cartilage is still unclear and controversial [3,4]. However, growing clinical and experimental evidence indicates that CaC crystals may induce real microcrystal stress on synoviocytes and chondrocytes, leading to OA lesion worsening and/or development [5].

Articular calcification is a well-known phenomenon observed in late-stage OA [6-8]. However, recent findings suggest that cartilage mineralization is a dynamic process occurring during or prior to OA development. Scotchford and colleagues detected BCP crystals in 12 cartilage samples from normal femoral heads of patients undergoing prosthetic replacement due to fracture of the femoral neck or distal femoral tumor. Transmission electron microscopy revealed calcifications in juvenile femoral head cartilage from patients as young as 10 years old and in five patients younger than 25 years old [9]. Similarly, Mitsuyama and colleagues, in a cadaveric study of 56 individual donors (mean age 50.3 years, range 12 to 74 years), showed calcifications in knee cartilage of young donors (23 donors <40 years old) and in normal-to-mildly affected cartilage, with 40% of tibial cartilage samples and 58% of femoral cartilage samples showing only grade 1 (macroscopically normal hyaline appearance) or grade 2 (minimal fibrillation) cartilage [10]. Moreover, the authors showed that calcification was diffused in the joint and comparable between femoral condyles and tibial plateaux. However, they did not distinguish superficial areas from deep zones, and the type of CaC crystals was not assessed [10]. Fuerst and colleagues reported that calcification was constantly identified in OA cartilage harvested during total knee replacement and that 40% of the mineralized cartilages were from patients with only mild OA lesions [6]. Moreover, chondrocytes isolated from OA cartilage showed differentiation toward a pro-mineralizing hypertrophic phenotype, whereas those isolated from normal cartilage did not [6]. Unfortunately, the authors examined only the most loaded and destroyed compartment of the knee joint, namely the medial femoral condyle cartilage. Sun and colleagues also showed that chondrocytes from OA meniscus could differentiate toward a pro-mineralized phenotype as compared with normal meniscus chondrocytes [11]. All of these findings suggest that hyaline and fibrous cartilage mineralization is a dynamic process involving chondrocyte metabolism dysfunction.

Extracellular inorganic phosphate (Pi) and extracellular inorganic pyrophosphate (PPi) concentrations are critical determinants of mineralization. Extracellular Pi and extracellular PPi are regulated by several proteins. The extracellular PPi concentration is controlled by three ectoenzymes: plasma-cell membrane glycoprotein 1 (PC-1)/ectonucleotide pyrophosphatase phosphodiesterase 1, which hydrolyzes nucleotide triphosphate into PPi; and AMP, the multipass transmembrane ankylosis (ANK) transporter; as well as the tissue-nonspecific alkaline phosphatases (TNAPs), which hydrolyze PPi into 2-Pi [12]. PPi is a potent inhibitor of apatite crystal formation, but its excess leads to CPP crystal deposition. The mRNA expression of these three molecules is higher in OA than in normal chondrocytes [11,13,14]. Sun and colleagues also showed increased expression of PC-1 and human ANK (ANKH) genes in chondrocytes isolated from OA meniscus [11].

We speculated that cartilage mineralization could affect the whole joint. We assessed the distribution of these mineral phases in human knee OA cartilage and their biochemical composition and morphological aspects, as well as changes in the expression of genes involved in the mineralization process. We found that cartilage calcification was a widespread phenomenon during OA, involving all joint compartments including nonweight-bearing zones and the whole depth of cartilage. Several types of CaC crystals were identified and the crystals displayed various morphological aspects. Finally, we observed that ANKH, PC-1 and TNAP expression was upregulated in chondrocytes isolated from CaC crystal-containing cartilages. Our results suggest that cartilage mineralization in OA may be associated with generalized chondrocyte dysfunction and changes toward a promineralizing phenotype.

## Materials and methods

### Patients

Specimens of knee joint cartilage were obtained from 20 prospective patients undergoing total knee replacement for OA at a single orthopedic department, Lariboisière hospital, Paris, France. One rheumatologist (CN) collected data on age, sex, body mass index, presence of genu varum or genu valgum deviation and preoperative knee plain radiographs (standard anteroposterior and lateral views) from the orthopedic charts of patients. Radiographs of the knees were analyzed for calcification deposition in medial and/or lateral joint spaces to define chondrocalcinosis. Knee OA radiographic severity was graded according to the Kellgren-Lawrence scale [15]. Patients with secondary OA (inflammatory disease or trauma) were excluded. In a second experiment, 14 different patients were included and knee joint cartilage was collected for histology studies and mRNA isolation.

### Cartilage sample preparation

**Figure 1:**
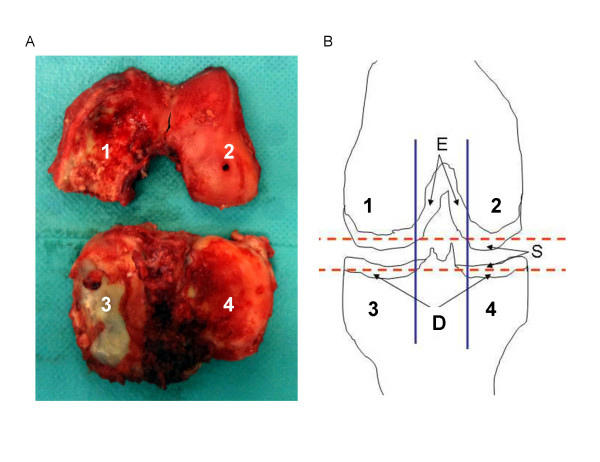
**Knee joint specimen obtained during total knee replacement and schematic representation of sample collection protocol**. **(A) **Specimens included femoral condyle and tibial plateau cartilage from both medial and lateral compartments, as previously described [12]. **(B) **Cartilage areas were: 1, medial condyle; 2, lateral condyle; 3, medial tibial plateau; 4, lateral tibial plateau; S, superficial layer; D, deep layer (two samples each of S and D); E, intercondyle cartilage was harvested if available. A maximum of 10 articular cartilage slices could be harvested from each knee joint.

We obtained specimens of femoral condyle and tibial plateau cartilage from both medial and lateral compartments (Figure 1A), for a theoretical total of eight samples of cartilage zones for each knee. Intercondylar cartilage was also retrieved when available (*n *= 9). Specimens were washed three times in calcium-free phosphate-buffered saline to avoid contamination by crystals released from subchondral bone at the time of surgery. Samples of each femoro-tibial compartment consisted of slices 1 mm thick, cut with a scalpel tangentially within the superficial and deep layers of the articular cartilage, as previously described (Figure 1B) [16]. Cartilage samples were directly frozen and stored at -20°C for scanning electron microscopy and Fourier-transform infrared (FT-IR) spectroscopy analysis. This protocol preserves the physico-chemical integrity of the mineral part of cartilage.

### Histology and immunohistochemistry staining

Cartilage samples were harvested from six different patients. For each patient, two adjacent cartilage samples were collected at the superficial zone of the medial and lateral femoral condyles and tibial plateaux. One sample was analyzed by FT-IR spectroscopy to identify the presence and type of CaC crystals. The second was fixed with 4% paraformaldehyde (pH 7.4) for 48 hours at 4°C, and then decalcified with 1% paraformaldehyde-0.2 M ethylenediamine tetraacetic acid (pH 7.4, 4°C). The specimens were then dehydrated with increasing concentrations of ethanol, before being embedded in paraffin. Paraffin sections (5 μm) were deparaffinized in Tissue Clear (Bayer Diagnostics, Puteaux, France) and rehydrated in a graded series of ethanol. Hydrogen peroxide (3%) was then used to quench endogenous peroxidase, and sections were incubated overnight in a pH 6.0 citrate buffer at 70°C. Nonspecific sites were blocked for 1 hour using the Impress Kit MP7500 (Vector/Vectastain, Les Ulis, France). Sections were then incubated overnight in humidified atmosphere at 4°C with primary polyclonal antibodies for ANK and ectonucleotide pyrophosphatase phosphodiesterase 1 (ENPP-1; 1:200) [17]. After two washes in phosphate-buffered saline, horseradish peroxidase-conjugated secondary antibody (goat anti-rabbit IgG, Vectastain ABC kit; Novocastra, Le Perray en Yvelines, France) was applied for 30 minutes at room temperature. The signal was developed with use of permanent AEC Chromogen (Diagomics, Blagnac, France). Nuclei were counterstained with hematoxylin, and slices were mounted in Eukitt (CML, Nemours, France). Sections incubated without primary antibody were controls.

For OA lesion analysis, sections were stained in 0.5% Safranin-O and rinsed with distilled water. OA lesion and immunohistochemistry (IHC) scoring was performed independently by two authors (H-KE and DC) with blinding to FT-IR spectroscopy results and other cartilage sample characteristics. Positive cells were counted on the same defined area and expressed as a percentage of the total cell count.

### RNA isolation and quantitative real-time reverse transcriptase-polymerase chain reaction

Chondrocytes were isolated from eight different tibial plateau samples of cartilage by enzymatic digestion (type 2 collagenase; Sigma, Saint-Quentin Fallavier, France) and placed into culture dishes for 30 minutes. Chondrocytes were then collected and the material that had deposited on the culture dish was harvested and centrifuged at 10,000 rpm for 10 minutes. The supernatant was discarded and pellets were dried and analyzed by FT-IR spectroscopy to detect the presence of CaC. Chondrocyte RNA was extracted using TRIzol (Roche, Paris, France). RNA was reverse transcribed into cDNAusing a High Capacity cDNA Reverse Transcription kit (Applied Biosystems, Barcelona, Spain), and then the cDNA product was amplified by polymerase chain reaction. The mRNA levels for ENPP1 (PC-1), ANKH, TNAP, solute carrier family 20 (SLC20A1) (Pit1), 5'-nucleotidase (ecto) (NT5E) (CD73) and ribosomal protein S29 were determined using the double strand-specific iTaq SYBR system (BioRad, Marnes-la-Coquette, France). All reactions involved the following thermal profile: 50°C for 2 minutes, denaturation at 95°C for 10 minutes; and then 40 cycles of denaturation at 95°C for 15 seconds, and annealing and extension at 60°C for 1 minute (data collection was performed at the end of each annealing/extension step) (StepOne Plus; Applied Biosystems). The third step ensures the specificity of the amplicons by measuring their melting temperature. Data analysis involved use of StepOne 2.1 (Applied Biosystems). Results were calculated by the 2^−ΔΔCt ^method.

The primer sequences were: ribosomal protein S29 (housekeeping gene), sense 5'-GGGTCACCAGCAGCTCGAGA-3' and anti-sense 5'-CAGACACGACAAGAGCGAGA-3'; ANKH, sense 5'-TGCTGTGTATCGTGCTTTCG-3' and anti-sense 5'-GAGTGACAGAGCCATGCAGA-3'; ENPP1 PC-1, sense 5'-TGGACCAGTCAGCAGTGAAG-3' and anti-sense 5'-TCAGGCATCTGTGCAAGTTC-3': TNAP, sense 5'-GCAGTGAAGGGCTTCTTGTC-3' and anti-sense 5'-CCACGTCTTCACATTTGGTG-3'; SLC20A1 (Pit1), sense 5'-CATGGTGGCAATGACGTAAG-3' and anti-sense 5'-TTGGTGTTGCCACTTTTGAA-3'; and NT5E (CD73), sense 5'-TTTTGCACACCAACGACGTG-3' and anti-sense 5'-GAACCTTGGTGAAGAGCCGA-3'.

### Fourier-transform infrared spectroscopy

The mineral phase was evaluated using a FT-IR Brucker Vector 22 spectrometer (Brucker Spectrospin, Wissembourg, France), with analysis by the KBr pellet method [18]. The ratio of the amount of sample mixed with the KBr was 1%. Data were collected in the absorption mode between 4,000 and 400/cm, with resolution 4/cm. The absorption bands of Ca^2+ ^biological reference compounds, namely CA, ACCP and CPP, are well assigned. For CA, the ν1 and ν3 P-O stretching vibration modes are measured at 960 to 962 and 1,035 to 1,045/cm, respectively, whereas the O-P-O ν4 bending mode corresponds to the doublet at 602 to 563/cm. The bands at 3,570 and 633/cm, corresponding to the stretching and vibrational modes, respectively, of the OH- groups, characteristic of hydroxyapatite, are absent for CA. The disappearance of the CA shoulder in the ν3 absorption band determines the presence of the ACCP compound. Regarding triclinic CPP, the O-P-O bending is recorded at 535 and 508/cm. The P-O stretching vibrations correspond to absorption at 923 and 991/cm, and the asymmetric stretching vibrations correspond to absorption at 1,037 and 1,089/cm.

### Scanning electron microscopy

A Zeiss SUPRA55-VP (Goettingen, Germany)field emission scanning electron microscope was used for microstructure observation. This field-effect gun microscope operates at 0.5 to 30 kV. High-resolution observations were obtained by two secondary electron detectors: in-lens SE and Everhart-Thornley SE detectors. To maintain the integrity of the specimens, measurements were performed at low voltage (approximately 2 kV) without the usual deposits of carbon at the surface of the sample. Prepared specimens were thawed, air dried and directly deposited on the sample holder.

### Statistical analysis

Data analysis involved using Systat 9 (SPSS Inc., Chicago, IL, USA). Quantitative variables are described with mean (standard error of the mean), unless indicated, and qualitative variables with number (percentage). Nonparametric tests were used because a normal distribution could not be demonstrated for all variables. Comparisons between groups involved the Fisher exact test for qualitative variables and the Mann-Whitney U test for quantitative data. *P *< 0.05 was considered statistically significant.

### Ethical considerations

All samples and experiments were collected and made in France. According to the French Ethical law on human research (L.12111-2 to L.1211-7, L.1211-2 and L.1245.2), informed consent and ethical approval were not necessary because tissues were surgical waste from routine joint replacement surgery and clinical data were anonymously treated. The investigation conformed to the principles of the Declaration of Helsinki.

## Results

### Demographic and clinical data

We included 20 OA patients (17 females). The mean (standard deviation) age and body mass index of patients at the time of total knee replacement was 74.6 (1.7) years (range 58 to 84 years) and 28.1 (1.6) kg/m^2^, respectively (Table 1). Preoperative plain radiographs (*n *= 18) detected unicompartmental, bicompartmental, or tricompartmental knee OA in two, eight, and eight patients, respectively. In all cases, OA was most severe in the medial femoro-tibial compartment, with a mean Kellgren-Lawrence score of 3.8 (0.1).

**Table 1:** Demographic, clinical, and osteoarthritis features for 20 patients undergoing total knee replacement

Characteristic	
Age (years)	74.6 (1.7)
Male gender	3/20 (15)
Body mass index (kg/m^2^)	28.1 (1.6)
Affected knee left/right	12/8
Genu varum	15/18 (83.3)
Kellgren-Lawrence grade	3.8 (0.1)
Grade 3	5/18 (22.2)
Grade 4	14/18 (77.8)
Unicompartmental osteoarthritis	2/18 (11.1)
Bicompartmental osteoarthritis	8/18 (38.9)
Tricompartmental osteoarthritis	8/18 (44.4)
Osteoarthritis mainly affecting the medial femoro-tibial compartment	18/18 (100)

Data presented as number of patients for whom data are available (%) or mean (standard deviation).

### Calcium-containing crystal prevalence and distribution in knee osteoarthritis

**Figure 2:**
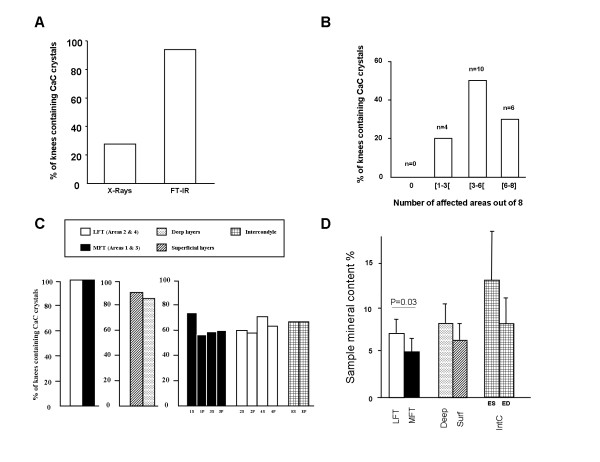
**Fourier-transform infrared spectroscopy of prevalence and localization of calcium-containing crystals in osteoarthritic knee articular cartilage**. **(A) **Data on preoperative knee joint radiographs (standard anteroposterior and lateral views) were retrieved from clinical charts for 18 of 20 included patients and assessed by one rheumatologist for presence of mineralization in the lateral and/or medial joint spaces to define chondrocalcinosis (first column) After surgery, specimens were harvested and analyzed by Fourier-transform infrared spectroscopy (FT-IR) to assess the presence and type of calcium-containing (CaC) crystals (second column). **(B) **FT-IR spectroscopy of cartilage samples harvested from the eight to 10 joint areas defined in Figure 1 from all 20 patients to determine the number of areas affected by calcification. n, number of patients. **(C) **Prevalence of calcifications and proportion of mineral content **(D) **as determined by FT-IR spectroscopy. Data are mean ± standard error of the mean. ED, intercondyle deep; ES, intercondyle superficial; IntC, intercondyle; LFT, lateral femoro-tibial compartment; MFT, medial femoro-tibial compartment; Surf, superficial.

Knee joint radiographs available for 18 of 20 patients showed calcification in the medial and/or lateral joint spaces in four (22.2%) cases, whereas FT-IR spectroscopy detected CaC crystals in specimens for all 20 patients (Figure 2A). Cartilage calcification was widespread. Among the eight assessed cartilage areas, CaC crystals were detected in one to three, three to six and six to eight areas for four (20%), 10 (50%) and six (30%) patients, respectively (Figure 2B). CaC crystal prevalence was similar between lateral and medial femoro-tibial compartments. In total, 90% and 85% of superficial and deep cartilage layers, respectively, contained CaC crystals (not statistically significant). CaC crystal prevalence ranged from 45% in the deep layer of the medial femoral condyle to 70% in the superficial layer of the medial femoral condyle, with no significant difference between superficial and deep areas (Figure 2C). Interestingly, CaC crystals were also detected in six of nine (66.6%) available intercondyle cartilage samples (Figure 2C).

The overall mean percentage of cartilage mineral content was higher in the lateral femoro-tibial than medial femoro-tibial compartment (7.3% (1.5) vs. 5.2% (1.5), *P *= 0.03), with no difference between the deep and superficial cartilage layers and different cartilage areas (Figure 2D).

### Calcium-containing crystal biochemical composition and morphological aspects

**Figure 3:**
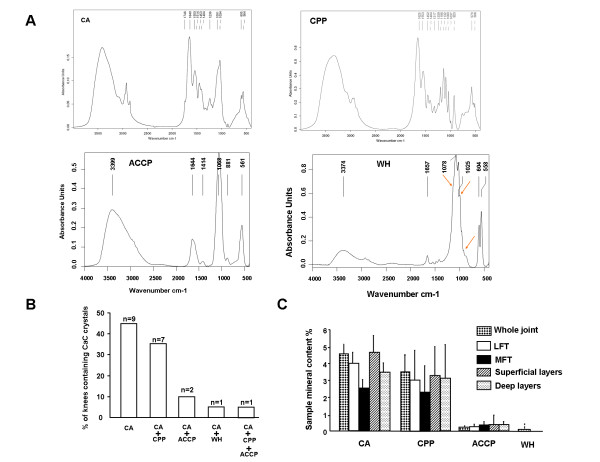
**Fourier-transform infrared spectroscopy of biochemical composition of calcium-containing crystals in osteoarthritic knee articular cartilage**. **(A) **Four distinct types of Fourier-transform infrared spectroscopy (FT-IR) spectra in osteoarthritis articular cartilage: carbonated apatite (CA) crystals, calcium pyrophosphate (CPP) crystals, amorphous Ca^2+ ^carbonated phosphate (ACCP) crystals and whitlockite (WH) crystals. **(B) **Joint-level prevalence of calcium-containing (CaC) crystal types. n, number of patients. **(C) **Content (%) of crystal type in samples according to joint compartment. Data are mean ± standard error of the mean. LFT, lateral femoro-tibial compartment; MFT, medial femoro-tibial compartment

Among 141 cartilage samples from 20 OA patients, FT-IR spectroscopy detected CaC in 63.8% (90/141) and revealed four distinct CaC crystal spectra: CA crystals in 71 samples, CPP crystals in 20 samples from eight patients, ACCP crystals in three samples from three patients, and WH crystals in four samples from the same patient (Figure 3A). CA crystals were the most frequently identified CaC crystal type and were detected in all OA knees (Figure 3B). They were the only crystal type in nine (45%) cases but were associated with CPP, ACCP and WH crystals in seven (35%) cases, three (15%) cases and one (5%) case, respectively, at the joint level. One patient had three types of calcium crystals, including CA, CPP and ACCP crystals. Remarkably, at the sample level (*n *= 141) CPP crystals were concomitantly detected in 16 of the 70 (22.8%) samples not containing CA crystals but in only four of the 71 (5.6%) samples containing CA crystals (*P *= 0.003) (Table 2). This difference was not found for the two other types of CaC crystals: ACCP and WH.

**Table 2:** Other calcium-containing crystal types in same cartilage area according to presence/absence of carbonated apatite crystals

Crystal	Samples without CA crystals (*n *= 70)	Samples with CA crystals (*n *= 71)	*P *value
CPP	16 (22.8)	4 (5.6)	0.003
ACCP	2 (2.9)	1 (1.4)	0.551
WH	1 (1.4)	3 (4.3)	0.317

Data presented as number (%). ACCP, amorphous carbonated calcium phosphate; CA, carbonated apatite; CPP, calcium pyrophosphate crystals; WH, whitlockite.

Among CaC crystals, CA crystals had a more diffuse spatial distribution and were identified in at least three of eight areas for 13 (65%) patients, whereas CPP, ACCP and WH crystals involved fewer than three areas when detected. The mean (standard error of the mean) overall mineral content of cartilage in knee joints was 7.7% (1.5). CA crystals were the most abundant CaC crystals in the knee OA joint as compared with CPP, ACCP and WH crystals (4.5% (0.6) vs. 3.1% (1.8), 0.3% (0.2), and 0.1% (0.1), respectively, *P *< 0.001) (Figure 3C). The amount of the four types of CaC was similar between medial and lateral femoro-tibial compartments and between superficial and deep cartilage layers (Figure 3C).

**Figure 4:**
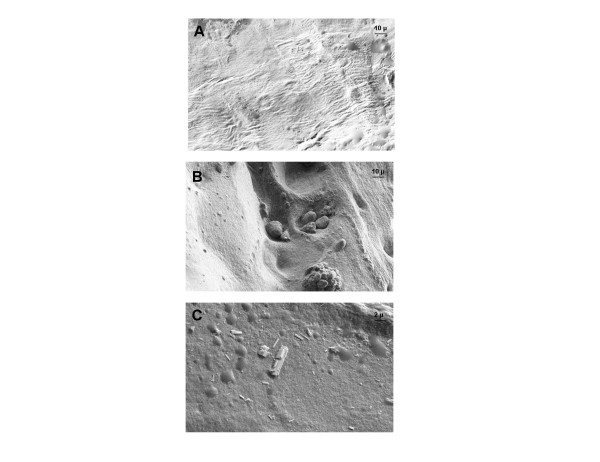
**Scanning electron microscopy of morphological aspects of calcium-containing crystals in osteoarthritic knee articular cartilage**. Representative images of crystals in cartilage. **(A) **Cartilage without calcification. (×755 magnification). **(B) **Agglomeration of several apatite crystallites localized in structure sets suggestive of chondrons and an agglomeration of apatite crystallites (×755 magnification). **(C) **Typical rod-shaped calcium pyrophosphate crystals and cubic morphologic feature of some crystallites (×2,999 magnification).

Scanning electron microscopy revealed two distinct morphological patterns according to CaC crystal biochemical composition. From a physico-chemical point of view, we used the terms 'nanocrystals' and 'crystallites' according to Van Meerssche and Feneau-Dupont [19] to define the structural hierarchy of these mineral concrements. Crystallites (measuring typically some tens of micrometers) are made of a collection of nanocrystals (measuring typically some tens or hundreds of nanometers) [19]. For CA crystallites, we found the same morphologic features as found in urinary calculi [20], resulting in an agglomeration of nanometer-scale apatite crystals and proteins (Figure 4B, C). Some of these structures were located near structure sets suggestive of chondrons, containing apatite crystallite deposits. For CPP crystals, scanning electron microscopy revealed typical rod-shaped crystallites of different sizes and shapes found widespread at the cartilage sample surface (Figure 4C). Some of these crystallites showed a cubic morphologic pattern.

### Clinical and radiological correlates with Ca^2+ ^crystal deposits

The CaC crystal amount in OA knee joints was not associated with age or body mass index. Mean cartilage CA crystal content in the whole joint was significantly higher in joints with chondrocalcinosis than without chondrocalcinosis as revealed by X-ray scans (6.0% (1.6) vs. 3.8% (2.0), *P *< 0.05). Conversely, joints with and without chondrocalcinosis did not differ in cartilage CPP or ACCP crystal content, whatever the location (whole joint, lateral or medial compartments) or the number of affected areas. These features were similar for patients with Kellgren-Lawrence grade 4 and grade < 4.

### Cartilage calcification is associated with increased expression of genes involved in mineralization process

**Figure 5:**
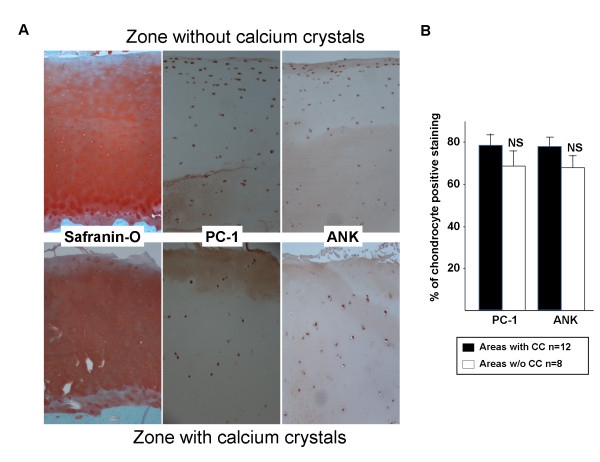
**Human plasma-cell-membrane glycoprotein 1 and homolog of progressive ankylosis expression in cartilage with/without calcification**. Examples of normal cartilage without calcification and mildly degraded cartilage containing carbonated apatite crystals. **(A) **Safranin-O staining and immunohistochemistry (IHC) staining of human plasma-cell-membrane glycoprotein 1 (PC-1) and homolog of progressive ankylosis (ANKH). **(B) **Quantitative expression of PC-1 and ANK according to the presence or not of calcium-containing crystals. Immunohistochemistry analysis was performed by two researchers who were blinded to Fourier-transform infrared spectroscopy results. The number of chondrocytes expressing PC-1 or ANKH is relative to the total number of chondrocytes. CC = calcium-containing crystals.

To assess the mechanisms by which the widespread calcification phenomenon in OA cartilage occurred, we performed IHC and analyzed the expression of several genes involved in the metabolism of PPi and Pi, including ENPP-1 (PC-1), ANKH, TNAP, SCL20A1 (Pit1) and NT5E (CD73). OA lesions were assessed by Safranin-O staining of 22 cartilage samples obtained from six different patients according to Osteoarthritis Research Society International recommendations [21]. Medial and lateral femoral condyles and tibial plateaux were analyzed. Eight of 22 (36.3%) samples did not display any OA lesions and 14 of 22 (63.6%) displayed grade 2 or grade 3 OA lesions (Figure 5A). The absence and mild OA lesions were due to the samples being harvested in zones where the cartilage was still visible macroscopically. This sampling was required for FT-IR spectroscopy analysis. CA crystals were detected in three of the eight (37.5%) cartilage samples without OA lesions and in seven of the 14 (50%) with OA lesions as determined by Safranin-O staining. The ratio of chondrocytes with positive staining for PC-1 or ANKH expression was similar between cartilage samples with and without CA crystals (77.2% (5.0) vs. 67.9% (6.2) for ANKH (*P *= 0.21), and 78.5% (5.6) vs. 68.3% (7.7) for PC-1 (*P *= 0.25), respectively) (Figure 5A, B).

To assess the whole tibial cartilage and not just a limited cartilage area, we analyzed the expression of genes involved in PPi and Pi metabolism and assessed their association with presence of CaC crystals. We isolated chondrocyte mRNA from the tibial plateau cartilage for eight new patients. Six samples contained calcification and two did not. CPP crystals were identified in 3/6 samples, CA crystals in 2/6 and both CPP and CA crystals in 1/6. The two samples without calcification were used as a control and the mRNA expression of the different proteins was relative to expression in these two samples. In cartilage samples containing CPP crystals, ENPP1 (PC-1) and ANKH mRNA levels were significantly higher, by 2.6-fold (*P *< 0.01) and 3.7-fold (*P = *0.05), respectively, than in cartilage without CPP crystals (Table 3), with no significant difference for levels of TNAP, SLCA201 (Pit1) and NT5E (CD73). Similarly, ENPP-1, TNAP and ANKH mRNA levels were higher in cartilage with CA crystals than in cartilage without CA crystals (1.81-fold, 5.73-fold and 2.24-fold, respectively), with no difference in levels of SLC20A1 (Pit1) and NT5E (CD73).

**Table 3:** Genes differentially expressed by cartilage calcification in samples from six patients with osteoarthritis

Gene name	Gene ID	Differential gene expression (fold-change) compared with OA cartilage without calcification							
		
		CPP crystals				CA crystals			CPP + CA
		
		OA1	OA2	OA3	Mean	OA4	OA5	Mean	OA6
ENPP1 (PC-1)	MIM 173335	2.98	2.29	2.59	2.62**	2.54	1.09	1.81 (NS)	2.5
ANKH	MIM 605145	3.54	2.67	4.94	3.72*	2.66	1.83	2.24 (NS)	1.82
TNAP	MIM 171760	2.20	7.74	56.10	22.01 (NS)	9.26	2.20	5.73 (NS)	2.17
SLC20A1 (Pit1)	MIM 137570	1.55	1.34	0.82	1.24 (NS)	1.82	1.63	1.72 (NS)	1.19
NT5E (CD73)	MIM 129190	0.82	1.27	0.78	0.96 (NS)	2.00	0.57	1.28 (NS)	1.59

ANKH, ankylosis progressive homolog; CA, carbonated apatite; CPP, calcium pyrophosphate; ENPP1, ectonucleotide pyrophosphatase 1; NS, not significant; NT5E, 5'-nucleotidase (ecto) or CD73 protein; OA, osteoarthritis; SLC20A1, solute carrier family 20 (phosphate transporter), member 1 or Pit1; TNAP, tissue nonspecific alkaline phosphatase. **P < 0.01 (vs. samples without calcification), *P < 0.05 (vs. samples without calcification).

## Discussion

Mineralization of hyaline cartilage and fibrocartilage is a common phenomenon of end-stage OA and has been associated with OA progression and cartilage destruction [6,11]. We confirmed the high frequency of cartilage mineralization in knee articular cartilage harvested during total knee replacement. Furthermore, cartilage mineralization was widespread in OA and occurred with similar frequency in all articular cartilage compartments, including nonweight-bearing zones and those free of lesions (such as intercondyle cartilage). In addition, the presence of CaC crystals was associated with a significant upregulation of the expression of genes, including ENPP1 (PC-1) and ANKH, which are involved in Pi and PPi homeostasis.

In a cadaveric study of 106 knees, Mitsuyama and colleagues showed that calcification was widespread in knee cartilages and involved both femoral condyles and tibial plateaus [10]. Similarly, in an anatomic, radiographic and magnetic resonance imaging study of 10 cadaveric knees of older subjects, Abreu and colleagues found diffuse calcifications involving menisci and hyaline cartilage but also other joint tissues such as cruciate ligaments, popliteus tendon and joint capsule in 40% of the joints [22]. Finally, in a cadaveric study of 68 knees, computed tomography frequently detected calcifications in menisci, femoro-tibial cartilages as well as proximal tibio-fibular cartilages [23]. However, all of these studies only assessed the presence of calcifications without characterizing the type of CaC crystals. Here, we used FT-IR spectroscopy and scanning electron microscopy to describe for the first time the distribution and type of CaC crystals in the whole knee joint, systematically analyzing eight to 10 cartilage areas for each knee, according to a standardized sample-harvesting protocol.

Although CA crystals were the main mineral detected, cartilage mineralization consisted of several types of CaC crystals with different physico-chemical properties and different shapes. As in Fuerst and colleagues' study, BCP crystals were detected in all samples whereas CPP crystals were found in only 40% [6]. However, the authors only analyzed the medial femoral condyle and did not differentiate superficial from deep zone cartilage. We rarely found CPP and BCP crystals in the same sample or zone, which suggests a tight balance between Pi and PPi metabolism. Indeed, excess PPi can lead to CPP crystal deposition, whereas low levels favor BCP crystal formation [24]. However, the observation of both CaC crystal types in some samples suggests that the Pi/PPi balance could change during OA disease.

The mechanisms of articular cartilage calcification in normal and OA cartilages are unknown. Several factors identified include genetics, aging, extracellular matrix modifications, imbalance between inhibitors and promineralizing factors, dysregulation of PPi and Pi metabolism, and chondrocyte phenotype alterations such as hypertrophic differentiation and apoptosis [25,26]. Several studies have shown that chondrocytes isolated from OA cartilage were prone to induce calcifications *in vitro*. The phenotype of these chondrocytes differed from that of chondrocytes isolated from normal cartilage. They were hypertrophic, expressed type X collagen and/or had upregulated expression of several genes involved in the mineralization process including ENPP-1 and ANKH [6,11,27]. We also observed an increase in mRNA expression of ENPP-1 (PC-1) and ANKH in chondrocytes from tibial cartilage containing CPP crystals as compared with cartilage without CPP crystals. However, IHC revealed no differences at the protein level. This result could be explained by the mild degradation of the cartilage samples used for IHC assessment. Further studies assessing more severe OA cartilage are required. Hirose and colleagues found a similar expression of PC-1 and ANK in OA cartilage with and without CPP crystal deposition [28]. However, extracellular PPi production was greater in chondrocytes from CPP crystal-containing cartilage than OA cartilage without crystals. These proteins may have both post-transcriptional and post-translational regulation mechanisms, but other proteins may be involved in the mineralization process. Recently, CD73 protein was found to have a potent inhibitor role in vascular and peri-articular mineralization [29]. St Hilaire and colleagues showed nonfunctional mutations in *NT5E *in members of three families with symptomatic arterial and joint calcifications. This gene encodes CD73, which participates in the extracellular pathway that converts AMP to adenosine. PC-1 hydrolyzes ATP into PPi and AMP, which is then converted to adenosine and Pi by CD73. Adenosine inhibits TNAP activity. CD73 deficiency therefore leads to increased TNAP activity, thus enhancing PPi degradation and Pi accumulation, both of which favor mineralization [29]. Whether the same phenomenon exists in OA cartilage needs to be further assessed. We observed that the NT5E mRNA expression was similar in chondrocytes isolated from cartilage with and without calcification. The protein level was not assessed because no efficient antibody is available.

In this study we did not show any association of radiographic OA grade or age and cartilage calcification, probably because our study was underpowered with its small patient sample and because our samples were mostly end-stage OA. However, Fuerst and colleagues showed that a radiographic Kellgren-Lawrence score and histological lesions correlated with cartilage calcification [6]. Moreover, Nalbant and colleagues reported that the presence of CaC crystals in OA synovial fluid was a predictor of OA progression [30]. Recently, we showed that intra-articular injection of BCP crystals into mouse knee induced cartilage degradation along with synovial inflammation [31]. Altogether, these data indicate that cartilage calcification may represent a significant pathogenic process during OA. Several issues remain to be addressed in CaC crystal-associated OA management, including the development of diagnostic tools to conveniently detect BCP crystals in synovial fluid and imaging techniques to detect CaC crystals in OA joints *in vivo *such as high-resolution computed tomography or ultrasonography, which could be used on a routine basis.

## Conclusions

Our results suggest that cartilage mineralization is a widespread and constant process in the OA joint, occurring even in nonweight-bearing intercondyle cartilage. Mineralization involved several compartments and several types of CaC crystals. This process is associated with an increase in the expression of genes involved in Pi and PPi homeostasis, which suggests a switch toward a promineralizing chondrocyte phenotype in OA. Molecular and biomechanical mechanisms controlling the articular chondrocyte fate toward a promineralizing phenotype and driving cartilage mineralization in OA remain largely undetermined. Further investigation with larger samples is required to address these questions and could lead to new specific therapies in OA targeting the associated mineralization process.

## Abbreviations

ACCP: amorphous Ca^2+ ^carbonated phosphate; ANKH: ankylosis progressive homolog; BCP: basic calcium phosphate; CA: carbonated apatite; CaC: calcium-containing; CPP: calcium pyrophosphate; ENPP1: ectonucleotide pyrophosphatase 1; IHC: immunohistochemistry; FT-IR: Fourier-transform infrared; NT5E: 5'-nucleotidase (ecto) or CD73 protein; OA: osteoarthritis; PC-1: plasma-cell membrane glycoprotein 1; Pi: inorganic phosphate; PPi: inorganic pyrophosphate; SLC20A1: solute carrier family 20; TNAP: tissue nonspecific alkaline phosphatase; WH: whitlockite.

## Competing interests

The authors declare that they have no competing interests.

## Authors' contributions

CN contributed to the study design, collection of cartilage samples, collection of clinical and radiological data, spectroscopy and microscopy studies, interpretation and analysis of data, and manuscript drafting. DB and MD carried out FT-IR spectroscopy and microscopy data acquisition, interpretation and analysis. AC-C contributed to collection of cartilage samples, was in charge of RNA isolation, and participated in the analysis of the data. DH was in charge of collection of cartilage samples and participated in the analysis of data. AB was in charge for IHC study, real-time reverse transcriptase quantitative polymerase chain reaction, and participated in the study design and analysis of data. DC participated in the collection of cartilage samples, histology and immunohistochemistry studies. AS and NB contributed to the conception and design of the study, analysis and interpretation of data and were involved in drafting the manuscript. FL and H-KE were involved in the conception and design of the study, interpretation and analysis of data, and drafting the manuscript. All authors read and approved the final manuscript.
